# Minimally invasive vs standard open carpal tunnel release: A systematic review, meta-analysis, and trial sequential analysis of randomized controlled trials

**DOI:** 10.1016/j.jpra.2026.08.018

**Published:** 2026-09-11

**Authors:** Ahmed Khaled Almarri, Yazan J Alalwani, Ammar Alsayegh, Mohammed Khder M. Alamri, Faisal Falah Alanazi, Abdulmalik Hadi AlEnazy, Daniyah Adil A. Alharbi, Nawaf Salman Almutairi, Ghayed Ahmad AlWassiah, Anna Abdulmohsen Almutlaq, Abdulmajeed Abdulaziz Alfouzan, Fadi Ibrahim Alhobayyeb, Raneem M. Turkistani, Abdulaziz Ibrahim Alzahrani, Ahmed Y Azzam

**Affiliations:** a Orthopedic Surgery Resident, Department of Orthopedic Surgery, College of Medicine, Imam Abdulrahman bin Faisal University, Dammam, Saudi Arabia; b College of Medicine, Imam Abdulrahman bin Faisal University, Dammam, Saudi Arabia; c Department of Orthopedic Surgery, East Jeddah General Hospital, Jeddah, Saudi Arabia; d College of Medicine, Sulaiman AlRajhi University, Bukayriyah, Saudi Arabia; e College of Medicine, Umm Al-Qura University, Makkah, Saudi Arabia; f College of Medicine, University of Jeddah, Jeddah, Saudi Arabia; g College of Medicine, Alfaisal University, Riyadh, Saudi Arabia; h College of Medicine, Qassim University, Qassim, Saudi Arabia; i Plastic and Reconstructive Surgery, King Abdulaziz Medical City, NGHA, Jeddah, Saudi Arabia; j Orthopedic Department, Buraydah Central Hospital, Qassim Cluster, Saudi Arabia; k Division of Global Health and Public Health, School of Nursing, Midwifery and Public Health, University of Suffolk, Ipswich, UK; l Clinical Research and Clinical Artificial Intelligence, ASIDE Healthcare, Lewes, Delaware, USA

**Keywords:** Carpal tunnel syndrome, Minimally invasive surgery, Carpal tunnel release, Median nerve, Trial sequential analysis

## Abstract

**Introduction:**

Carpal tunnel syndrome (CTS) is the most common peripheral nerve entrapment disorder around the world. Minimally invasive carpal tunnel release (MI-OCTR) techniques have been developed as alternatives to standard open CTR; however, the evidence comparing these approaches remains inconclusive. Our study aimed to evaluate the efficacy, safety, and perioperative outcomes of MI-OCTR versus standard open CTR.

**Methods:**

Following PRISMA 2020 guidelines, PubMed, Scopus, Web of Science, Cochrane Library, and Google Scholar were searched from inception through 20 December 2025. Randomized controlled trials (RCTs) comparing MI-OCTR with standard open CTR were included. Random-effects meta-analysis with Hartung–Knapp–Sidik–Jonkman (HKSJ) adjustment and trial sequential analysis (TSA) were performed.

**Results:**

Ten RCTs (660 randomized units) were included. No significant differences were observed for Boston Carpal Tunnel Questionnaire Symptom Severity Scale (BCTQ-SSS), (mean difference [MD] = −0.36, 95% confidence interval [CI]: −1.07 to 0.35, P-value = 0.23, I² = 98.2%) or Functional Status Scale (MD = −0.27, 95% CI: −0.75 to 0.21, P-value = 0.19). MI-OCTR demonstrated significantly shorter incision length (MD = −2.00 cm, P-value = 0.04) and earlier return to work (MD = −9.66 days, P-value = 0.02). TSA confirmed that only 1.2% of the required information size for BCTQ-SSS had been accrued, indicating the evidence remains inconclusive.

**Conclusions:**

MI-OCTR and standard open CTR demonstrated similar efficacy in symptom relief, with MI-OCTR offering possible perioperative advantages. The certainty of evidence was very low for most outcomes, and TSA indicated that significantly larger trials are needed to reach definitive conclusions.

## Introduction

Carpal tunnel syndrome (CTS) is the most common peripheral nerve entrapment disorder around the world, with an estimated prevalence of up to 5% in the general population depending on the diagnostic criteria utilized.^1^^,^^2^ The condition results from compression of the median nerve within the carpal tunnel at the wrist, leading to pain, numbness, and tingling in the affected hand, which significantly impacts quality of life and functional capacity. CTS has been shown to demonstrate a higher incidence among women, with peak occurrence between the ages of 50 and 54 years, and it represents a significant cause of work disability around the world.^3^^,^^4^ A recent meta-analysis investigating over five million individuals across 31 prevalence studies reported a pooled estimate of 14.4%, further highlighting the significant burden of this condition on healthcare systems.^5^

When conservative measures fail to provide adequate relief, carpal tunnel release (CTR) is considered the definitive surgical management for CTS. The standard open CTR technique involves a longitudinal palmar incision of around three to four centimeters to directly visualize and divide the transverse carpal ligament.^6^ However, the standard open approach has been associated with possible complications including pillar pain, scar tenderness, prolonged grip weakness, and delayed return to work, which have motivated the development of minimally invasive alternatives.^7^^,^^8^ Various minimally invasive CTR (MI-OCTR) techniques have been introduced, including mini-open incisions, endoscopic single-portal and dual-portal approaches, Knifelight-assisted release, and ultrasound-guided procedures, all of which aim to reduce surgical trauma while achieving complete division of the transverse carpal ligament.^9^^,^^10^

Previous studies investigated and discussed specific comparisons within carpal tunnel surgery, including endoscopic versus open release,^11^^,^^12^ mini-transverse versus longitudinal techniques,^13^ and pillar pain outcomes after different approaches.^14^ However, prior studies some of them have been limited by focusing on individual MI technique subtypes rather than evaluating the full spectrum of MI-OCTR approaches as a collective intervention against standard open CTR. In addition to that, no structured meta-analysis to date has used trial sequential analysis (TSA) to evaluate whether the accumulated evidence has reached sufficient statistical power to support definitive conclusions regarding MI-OCTR efficacy and safety.

To address these evidence gaps, our study aimed to conduct a structured systematic review and meta-analysis with TSA of all available randomized controlled trials (RCTs) comparing MI-OCTR techniques with standard open CTR. The primary outcomes investigated were symptom severity and functional status measured by the Boston Carpal Tunnel Questionnaire (BCTQ),^15^ while secondary outcomes included scar morbidity, perioperative parameters, and safety. We hypothesized that MI-OCTR techniques would demonstrate comparable efficacy to standard open CTR while offering advantages in perioperative outcomes.

## Methods

### Study design and search strategy

Our study was conducted in accordance with the Preferred Reporting Items for Systematic Reviews and Meta-Analyses (PRISMA) 2020 guidelines.^16^^,^^17^ A structured literature search was performed across five electronic databases, including PubMed, Scopus, Web of Science, Cochrane Library, and Google Scholar, from inception through 20 December 2025. The search strategy utilized Medical Subject Headings (MeSH) terms and free-text keywords related to the population (carpal tunnel syndrome, median nerve entrapment), intervention (minimally invasive, mini-open, limited incision, endoscopic, Knifelight, ultrasound-guided), comparison (open release, standard release), and outcome (symptom severity, functional status, complications, return to work), combined with Boolean operators "AND" and "OR." Reference lists of included studies and relevant reviews were manually screened. The search was limited with focusing on English-language based studies only.

### Eligibility criteria

Studies were included if they were: (1) RCTs; (2) enrolled adults diagnosed with CTS; (3) compared any MI-OCTR technique with standard open CTR utilizing a traditional longitudinal palmar incision; and (4) reported at least one quantitative outcome. Studies were excluded if non-randomized, compared two MI techniques without a standard open control, included revision surgery only, or lacked extractable outcome data.

### Study selection and data extraction

Titles and abstracts were screened independently by two reviewers, with disagreements resolved through consensus. Full-text reports were then assessed against inclusion criteria. Data extraction was performed to extract study characteristics, patient demographics, intervention details, and all outcomes including BCTQ Symptom Severity Scale (BCTQ-SSS), BCTQ Functional Status Scale (BCTQ-FSS), scar morbidity measures (pillar pain, scar tenderness), perioperative parameters (operative time, incision length, return to work, grip strength), and safety outcomes (total complications, nerve injury, symptom recurrence, scar hypertrophy).

### Risk of bias assessment

The methodological quality of included RCTs was evaluated utilizing the Cochrane Risk of Bias 2 (RoB 2) tool across five domains: randomization process (D1), deviations from intended interventions (D2), missing outcome data (D3), measurement of the outcome (D4), and selection of the reported result (D5), with overall judgment following the RoB 2 scale and its algorithm for assessment.^18^

### Statistical analysis

All analyses were conducted using RStudio statistical software version 2025.09 with R version 4.4.2. The inverse-variance random-effects model (DerSimonian–Laird τ² estimator) with Hartung–Knapp–Sidik–Jonkman (HKSJ) adjustment was applied for all pooled analyses. Mean difference (MD) was utilized for continuous outcomes, standardized mean difference (SMD) for outcomes with heterogeneous units, and odds ratio (OR) for binary outcomes. Heterogeneity was quantified with the Cochran Q test, I² statistic, τ², and 95% prediction intervals. Subgroup analyses were performed by MI technique type and follow-up timepoint with interaction Q tests. Leave-one-out sensitivity analysis, influence diagnostics, and pre-specified sensitivity analyses were conducted. Univariate meta-regression (method of moments) was performed for outcomes with k ≥ 5. Publication bias was evaluated through funnel plots, Egger's regression test for continuous outcomes, Peters' regression test for binary outcomes, Begg's rank correlation test, Duval and Tweedie trim-and-fill adjustment method, and the Rücker limit meta-analysis.

### Trial sequential analysis

TSA was conducted with two-sided α = 0.05 and 80% power.^19^, ^20^, ^21^ Minimal clinically important differences (MCIDs) were set at MD = 0.30 for BCTQ-SSS/FSS, MD = 1.0 cm for incision length, MD = 7.0 days for return to work, and OR = 0.50 for binary outcomes. The required information size (RIS) was calculated adjusting for heterogeneity (D²), with O'Brien–Fleming monitoring boundaries and β-spending futility boundaries.

### Certainty of evidence

The certainty of evidence was evaluated utilizing the Grading of Recommendations Assessment, Development, and Evaluation (GRADE) framework,^22^^,^^23^ starting at high for RCTs and downgraded across risk of bias, inconsistency, indirectness, imprecision, and publication bias.

## Results

### Study selection

**Fig. 1:**
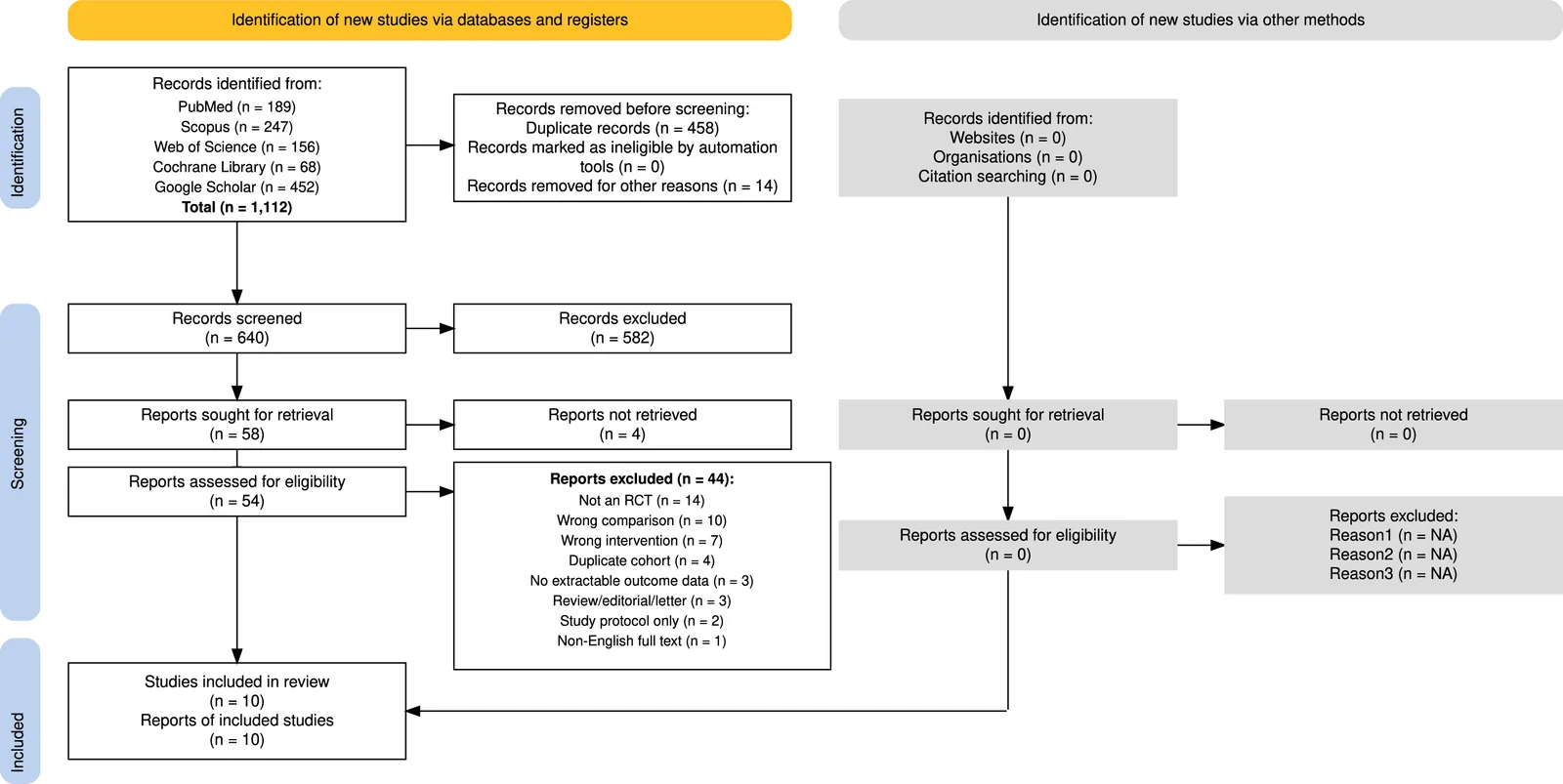
PRISMA flow diagram.

The database search resulted 1112 records (PubMed 189, Scopus 247, Web of Science 156, Cochrane Library 68, Google Scholar 452). After removing 458 duplicates and 14 records for other reasons, 640 records were screened, of which 582 were excluded. Of 58 reports sought for retrieval, four were not retrieved. Among 54 full-text reports assessed, 44 were excluded: not an RCT (n = 14), wrong comparison (n = 10), wrong intervention (n = 7), duplicate cohort (n = 4), no extractable outcome data (n = 3), review/editorial/letter (n = 3), study protocol only (n = 2), and non-English full text (n = 1). Ten RCTs were included (Fig. 1).

### Study characteristics

The ten included RCTs were published between 2002 and 2025 across nine countries (India, China, Italy, the United States, Iran, Denmark, Thailand, the United Kingdom, and Croatia), enrolling 331 MI-OCTR and 329 standard open randomized units (Table 1). Mean age ranged from around 41 to 65 years, and female proportion from 50.0% to 93.3%. Seven studies utilized mini-open techniques, two utilized Knifelight, and one utilized endoscopic release. Follow-up ranged from three weeks to 36 months. Five studies reported baseline BCTQ scores, which were comparable between groups (Table 1).

**Table 1:** Baseline demographics and characteristics of included studies.

**Study**	**Country**	**Design**	**Number (MI / Std)**	**MI Technique**	**Age, years (MI / Std)**	**Female, n (%) (MI / Std)**	**Baseline BCTQ-SSS (MI / Std)**	**Baseline BCTQ-FSS (MI / Std)**	**Follow-up**
Gupta et al.^30^	India	RCT	30 / 30^h^	Transverse mini-port incision	43.3 ± 8.4 / 41.1 ± 7.7	26 (86.7) / 27 (90.0)	2.75 ± 0.24 / 2.80 ± 0.24	2.60 ± 0.29 / 2.61 ± 0.31	9 months
Zhang et al.^31^	China	RCT	73 / 65	Double small incisions	46 (33–65)^†^ / 45 (33–63)^†^	50 (68.5) / 43 (66.2)	3.70 ± 0.58 / 3.80 ± 0.62	3.20 ± 0.71 / 3.20 ± 0.71	36 months
Tarallo et al.^25^	Italy	RCT	60 / 60	Minimal access CTR	63 (56–75)^†^ / 65 (54–74)^†^	30 (50.0) / 30 (50.0)	3.90 ± 0.40 / 3.80 ± 0.50	3.70 ± 0.30 / 3.60 ± 0.40	12 months
Castillo and Yao^32^	USA	RCT	13 / 16^h^	Two-incision (transverse wrist + longitudinal palm)	62.0 ± 14.1 / 62.9 ± 17.2	9 (69.2) / 12 (75.0)	2.91 ± 0.59 / 2.84 ± 0.77	2.54 ± 0.75 / 2.39 ± 0.97	6 months
Heidarian et al. ^a,^^33^	Iran	RCT	29 / 30	Knifelight release	49.7 ± 7.9 / 45.5 ± 10.2	25 (86.2) / 28 (93.3)	NR	NR	3 weeks
Larsen et al. ^b,^^34^	Denmark	RCT^‡^	30 / 30	Endoscopic single-portal (Menon technique)	54 (33–80)^†^ / 54 (28–83)^†^	22 (73.3) / 18 (60.0)	NR	NR	6 months
Suppaphol et al.^35^	Thailand	RCT	15 / 15^h^	Limited open (tunneling)	53.3 / 53.1^§^	14 (93.3) / 13 (86.7)	3.20 / 2.87^§^	2.95 / 2.96^§^	3 months
Hamed et al.^36^	UK	RCT	19 / 21	Double-incision	54 ± 8 / 49 ± 9.6	15 (78.9) / 16 (76.2)	NR	NR	6 months
Bhattacharya et al. ^a,^^37^	UK	RCT^‖^	26 / 26^h^	Knifelight release	48^§‖^	18 (69.2)^‖^	NR	NR	6 weeks
Jugovac et al.^38^	Croatia	RCT	36 / 36	Limited palmar incision (microscope-assisted)	54.2 ± 8.9 / 52.5 ± 9.7	31 (86.1) / 23 (63.9)	NR	NR	3 months

**Notes:** Values are presented as mean ± SD unless otherwise indicated; MI / Std denotes the minimally invasive group and standard open group, respectively; ^a^Knifelight technique; ^b^Endoscopic technique; all unmarked studies used mini-open incision techniques; ^†^Median (range); ^‡^Three-arm trial, only endoscopic versus classic open arms extracted; ^§^SD not reported; ^‖^Within-patient split-body design, demographics represent the same patients in both groups; ^h^Unit of analysis is hands. **Abbreviations:** BCTQ = Boston Carpal Tunnel Questionnaire; CTR = Carpal Tunnel Release; FSS = Functional Status Scale; MI = Minimally Invasive; NR = Not Reported; RCT = Randomized Controlled Trial; SD = Standard Deviation; SSS = Symptom Severity Scale; Std = Standard Open.

### Risk of bias

Six of ten trials were rated high risk of bias overall, driven by Domain 2 (deviations from intended interventions), as participant and surgeon blinding are inherently impossible in surgical trials comparing different incision approaches. Four studies ^32^^,^^34^^,^^36^^,^^37^ were rated some concerns, having achieved patient blinding through standardized dressings or within-patient designs. Domain 3 (missing outcome data) was rated low risk in eight of ten studies, with some concerns in two ^32^^,^^34^ due to moderate attrition **(Supplementary Table 1)**.

### Primary efficacy outcomes

**Fig. 2:**
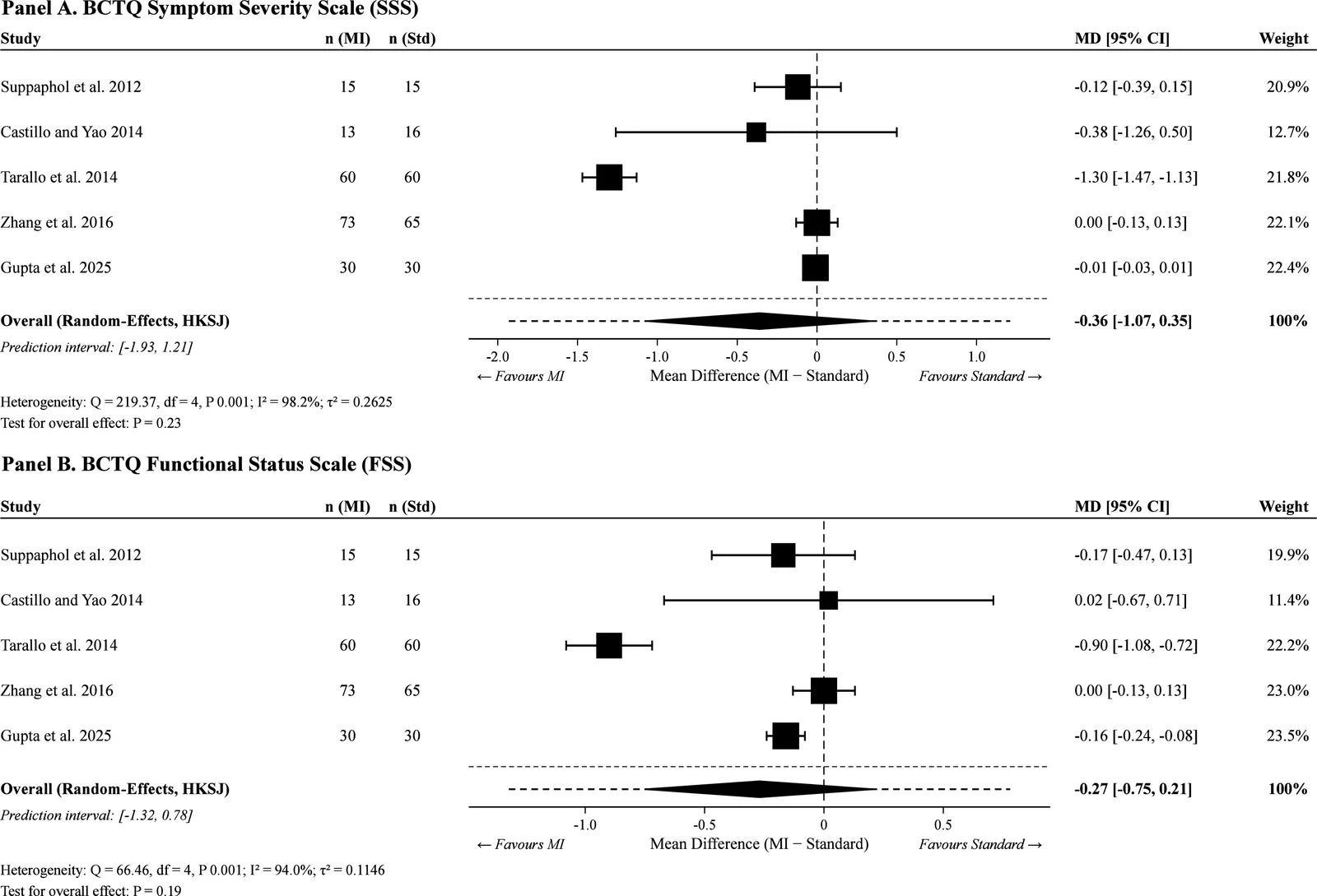
Forest plot for BCTQ-SSS and BCTQ-FSS.

Five studies (191 MI-OCTR, 186 standard open) reported BCTQ-SSS (Table 2). The pooled analysis demonstrated no significant difference (MD = −0.36, 95% CI: −1.07 to 0.35, P-value = 0.23), with very significant heterogeneity (I² = 98.2%, τ² = 0.2625, Cochran Q = 219.37, P-value<0.001) and a 95% prediction interval of −1.93 to 1.22 crossing the null (Fig. 2**, Panel A)**. Also, BCTQ-FSS showed no significant difference (MD = −0.27, 95% CI: −0.75 to 0.21, P-value = 0.19, I² = 94.0%, τ² = 0.1146, Cochran Q = 66.46, P-value<0.001; 95% PI: −1.31 to 0.78) (Fig. 2**, Panel B)**.

**Table 2:** Pooled meta-analytic results for all outcomes.

**Outcome**	**k**	**Participants (MI / Std)**	**Effect Measure**	**Pooled Estimate (95% CI)**	**P-value**	**I², %**	**τ²**	**Cochran Q (P-value)**	**95% PI**
**Primary Efficacy Outcomes:**									
BCTQ-SSS	5	191 / 186	MD	−0.36 (−1.07 to 0.35)	0.23	98.2	0.2625	219.37 (P = <0.001)	−1.93 to 1.22
BCTQ-FSS	5	191 / 186	MD	−0.27 (−0.75 to 0.21)	0.19	94.0	0.1146	66.46 (P = <0.001)	−1.31 to 0.78
**Scar Morbidity Outcomes:**									
Pillar Pain (early ≤6 wk)	2^†^	45 / 47	OR	0.36 (0.00–23,744.4)	0.45	71.2	1.088	3.47 (P = 0.06)	—
Pillar Pain (late ≥3 mo)	4	182 / 176	OR	0.78 (0.13–4.65)	0.69	58.8	0.7302	7.27 (P = 0.06)	0.03–20.15
Scar Tenderness (early ≤3 mo)	3	81 / 83	OR	0.24 (0.05–1.23)	0.06	0	0	0.31 (P = 0.86)	0.05–1.23
Scar Tenderness (late ≥6 mo)	3	152 / 146	OR	0.77 (0.14–4.31)	0.57	0	0	1.86 (P = 0.39)	0.14–4.31
**Perioperative Outcomes:**									
Operative Time, min	3	80 / 81	MD	−2.50 (−9.20 to 4.19)	0.25	96.2	6.0642	52.27 (P = <0.001)	−14.82 to 9.81
Incision Length, cm	3	80 / 81	MD	−2.00 (−3.77 to −0.22)	0.04	97.0	0.3885	67.64 (P = <0.001)	−5.11 to 1.12
Grip Strength	3	47 / 52	SMD	0.36 (−1.76 to 2.47)	0.54	80.1	0.5331	10.04 (P = 0.007)	−3.38 to 4.09
Return to Work, days	3	132 / 125	MD	−9.66 (−16.04 to −3.29)	0.02	67.2	2.5194	6.09 (P = 0.05)	−18.11 to −1.22
**Safety Outcomes:**									
Total Complications	3	116 / 116	OR	1.04 (0.06–17.54)	0.96	23.1	0.4294	2.6 (P = 0.27)	0.02–56.25
Nerve Injury	2^†^	49 / 51	OR	4.37 (0.00–8777,169.0)	0.42	0	0	0.04 (P = 0.85)	—
Symptom Recurrence	3	152 / 146	OR	0.58 (0.02–14.72)	0.55	0	0	0.27 (P = 0.87)	0.02–14.72
Scar Hypertrophy	2^†^	90 / 90	OR	0.11 (0.00–96,929.2)	0.29	0	0	0.21 (P = 0.65)	—

**Notes:** All analyses used the inverse-variance random-effects model (DerSimonian–Laird estimator for τ²) with Hartung–Knapp–Sidik–Jonkman adjustment for confidence intervals; negative MD values and OR < 1.00 favor the MI group; SSS and FSS pooled at the nearest available timepoint to three months per study (Suppaphol one month, Castillo six weeks, Tarallo six months, Zhang three years, Gupta 12 weeks for SSS and six weeks for FSS); Suppaphol three-month SSS excluded from primary analysis due to suspected SD reporting error in the control group; grip strength analyzed as SMD due to heterogeneous reporting units; return to work includes Heidarian return-to-ADL endpoint; studies with zero events in both arms excluded from OR calculations; ^†^k = 2, HKSJ adjustment yields very wide CI due to limited degrees of freedom, interpret with caution. **Abbreviations:** BCTQ = Boston Carpal Tunnel Questionnaire; CI = Confidence Interval; FSS = Functional Status Scale; I² = Higgins inconsistency index; k = number of studies; MD = Mean Difference; MI = Minimally Invasive; OR = Odds Ratio; PI = Prediction Interval; SMD = Standardized Mean Difference; SSS = Symptom Severity Scale; Std = Standard Open; τ² = between-study variance.

### Scar morbidity outcomes

**Fig. 3:**
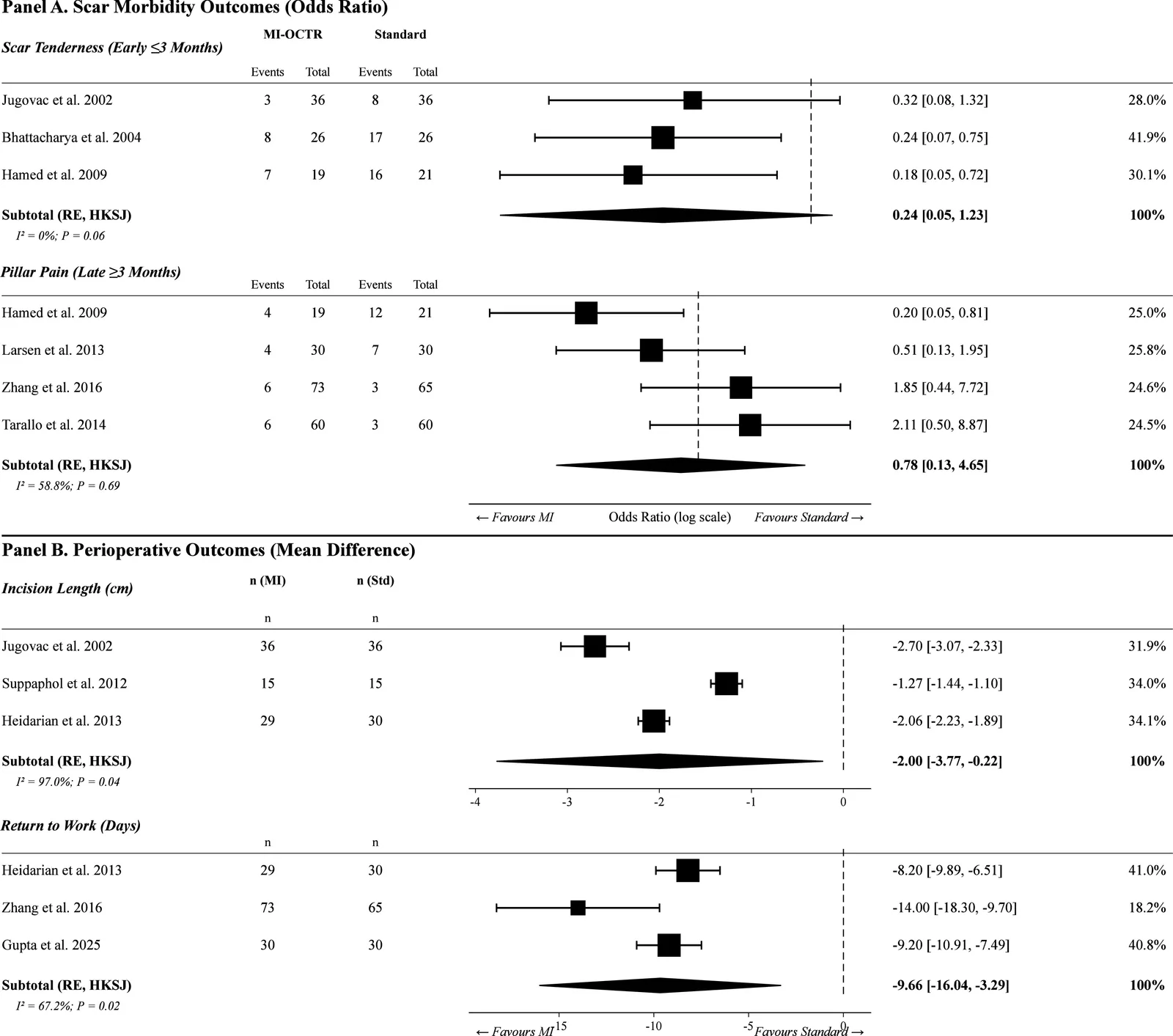
Forest plot for scar morbidity outcomes and perioperative outcomes.

Early scar tenderness (≤three-months, k = 3, 81/83) showed a non-significant trend favoring MI-OCTR (OR = 0.24, 95% CI: 0.05–1.23, P-value = 0.06, I² = 0%) (Fig. 3**, Panel A)**. Late scar tenderness (≥six-months, k = 3, 152/146) was non-significant (OR = 0.77, 95% CI: 0.14–4.31, P-value = 0.57, I² = 0%). Early pillar pain (≤six-weeks, k = 2, 45/47) showed no significant difference (OR = 0.36, 95% CI: 0.00–23,744.4, P-value = 0.45, I² = 71.2%), with the HKSJ adjustment resulting in a very wide 95% CI due to k = 2. Late pillar pain (≥three-months, k = 4, 182/176) was also non-significant (OR = 0.78, 95% CI: 0.13–4.65, P-value = 0.69, I² = 58.8%) (Table 2).

### Perioperative outcomes

MI-OCTR demonstrated significantly shorter incision length (k = 3, 80/81; MD = −2.00 cm, 95% CI: −3.77 to −0.22, P-value = 0.04, I² = 97.0%) and significantly earlier return to work (k = 3, 132/125; MD = −9.66 days, 95% CI: −16.04 to −3.29, P-value = 0.02, I² = 67.2%) (Fig. 3**, Panel B)**. Operative time (k = 3, 80/81; MD = −2.50 min, 95% CI: −9.20 to 4.19, P-value = 0.25, I² = 96.2%) and grip strength (k = 3, 47/52; SMD = 0.36, 95% CI: −1.76 to 2.47, P-value = 0.54, I² = 80.1%) were not significantly different (Table 2).

### Safety outcomes

No significant differences were observed for total complications (k = 3, 116/116; OR = 1.04, 95% CI: 0.06–17.54, P-value = 0.96), nerve injury (k = 2, 49/51; OR = 4.37, 95% CI: 0.00–8777,169.0, P-value = 0.42), symptom recurrence (k = 3, 152/146; OR = 0.58, 95% CI: 0.02–14.72, P-value = 0.55), or scar hypertrophy (k = 2, 90/90; OR = 0.11, 95% CI: 0.00–96,929.2, P-value = 0.29) (Table 2).

### Subgroup analyses

For BCTQ-SSS and BCTQ-FSS, all five studies utilized mini-open techniques, precluding between-technique comparison (Table 3). No significant interactions were observed between MI technique subgroups for any outcome: late pillar pain (mini-open OR = 0.92 vs endoscopic OR = 0.51; interaction P-value = 0.75), early scar tenderness (interaction P-value = 1.00), operative time (interaction P-value = 0.28), incision length (interaction P-value = 0.99), or return to work (interaction P-value = 0.85). Temporal subgroup analysis showed a trend from borderline early scar tenderness reduction (OR = 0.24, P-value = 0.06) toward attenuation at late follow-up (OR = 0.77, P-value = 0.57), without significant interaction (P-value = 0.33) (Table 3).

**Table 3:** Subgroup analyses by MI technique type and follow-up timepoint.

**Outcome**	**Subgroup**	**k**	**Participants (MI / Std)**	**Effect Measure**	**Pooled Estimate (95% CI)**	**P-value**	**I², %**	**Interaction Q (P-value)**
**Panel A - Subgroup by MI Technique Type:**								
**BCTQ-SSS**	Mini-Open (all 5 studies)	5	191 / 186	MD	−0.36 (−1.07 to 0.35)	0.23	98.2	—
**BCTQ-FSS**	Mini-Open (all 5 studies)	5	191 / 186	MD	−0.27 (−0.75 to 0.21)	0.19	94.0	
**Pillar Pain (late)**	Mini-Open	3	152 / 146	OR	0.92 (0.03–25.12)	0.92	70.4	0.1 (P = 0.75)
	Endoscopic	1^§^	30 / 30	OR	0.51 (0.13–1.95)	0.32	—	
**Scar Tenderness (early)**	Mini-Open	2	55 / 57	OR	0.24 (0.00–142.2)	0.21	0	0.0 (P = 1.00)
	Knifelight	1^§^	26 / 26	OR	0.24 (0.07–0.75)	0.01	—	
**Operative Time, min**	Mini-Open	2	51 / 51	MD	−1.18 (−8.90 to 6.54)	0.30	55.7	1.18 (P = 0.28)
	Knifelight	1^§^	29 / 30	MD	−5.50 (−6.50 to −4.50)	<0.001	—	
**Incision Length, cm**	Mini-Open	2	51 / 51	MD	−1.98 (−11.06 to 7.11)	0.22	97.9	0.0 (P = 0.99)
	Knifelight	1^§^	29 / 30	MD	−2.06 (−2.23 to −1.89)	<0.001	—	
**Return to Work, days**	Mini-Open	2	103 / 95	MD	−11.18 (−41.20 to 18.84)	0.13	75.8	0.04 (P = 0.85)
	Knifelight	1^§^	29 / 30	MD	−8.20 (−9.89 to −6.51)	<0.001	—	
**Panel B - Subgroup by Follow-up Timepoint:**								
**Pillar Pain**	Early (≤6 wk)	2	45 / 47	OR	0.36 (0.00–23,744.4)	0.45	71.2	0.0 (P = 1.00)
	Late (≥3 mo)	4	182 / 176	OR	0.78 (0.13–4.65)	0.69	58.8	
**Scar Tenderness**	Early (≤3 mo)	3	81 / 83	OR	0.24 (0.05–1.23)	0.06	0	0.95 (P = 0.33)
	Late (≥6 mo)	3	152 / 146	OR	0.77 (0.14–4.31)	0.57	0	
**Grip Strength**	Early (6 wk)	2	32 / 37	SMD	−0.01 (−6.99 to 6.98)	0.99	79.4	—
	Mid-term (3 mo)	2	34 / 36	SMD	0.70 (−4.13 to 5.53)	0.32	56.0	

**Notes:** Panel A stratifies outcomes by MI technique type (Mini-Open, Knifelight, or Endoscopic); Panel B stratifies outcomes by follow-up timepoint to assess temporal evolution of treatment effects; all pooled estimates use the inverse-variance random-effects model with HKSJ adjustment; interaction Q tests the null hypothesis of no difference between subgroups using a fixed-effect interaction Chi-squared test; for BCTQ-SSS and BCTQ-FSS only Mini-Open studies reported these outcomes hence no between-technique comparison was feasible; negative MD and OR < 1.00 favor the MI group; ^§^single-study estimate, no pooling performed, 95% CI from individual study. **Abbreviations:** BCTQ = Boston Carpal Tunnel Questionnaire; CI = Confidence Interval; FSS = Functional Status Scale; I² = Higgins inconsistency index; k = number of studies; MD = Mean Difference; MI = Minimally Invasive; OR = Odds Ratio; SMD = Standardized Mean Difference; SSS = Symptom Severity Scale; Std = Standard Open.

### Sensitivity analyses and meta-regression

Leave-one-out analysis demonstrated that no study removal reversed the direction of any pooled estimate (Table 4**, Panel A)**. Removing^25^ reduced BCTQ-SSS I² from 98.2% to 0.0% (revised MD = −0.01, 95% CI: −0.05 to 0.02) and BCTQ-FSS I² from 94.0% to 30.0%. Influence diagnostics confirmed^25^ as an outlier and influential for both outcomes (studentized residuals: −2.05 and −2.04; Cook's distances: 2.428 and 5.241) (Table 4**, Panel B)**. Pre-specified sensitivity analyses excluding studies based on design or technique did not alter conclusions (Table 4**, Panel C)**. Meta-regression identified publication year as a significant moderator for BCTQ-SSS (β = 0.0442, P-value = 0.003, R² = 37.9%), with total sample size (P-value = 0.006, R² = 21.1%) and MI incision length (P-value = 0.02, R² = 10.5%) also significant; follow-up duration was not (P-value = 0.55). No moderators were significant for BCTQ-FSS (Table 4**, Panel D)**. Cumulative meta-analysis showed the BCTQ-SSS estimate never reached significance, with I² rising from 0% to 96.3% upon addition of Tarallo et al.^25^ **(Supplementary Table 2, Panel A)**.

**Table 4:** Sensitivity analyses, influence diagnostics, and meta-regression.

**Panel A - Leave-One-Out Sensitivity Analysis:**							
**Outcome**	**Study Removed**	**k**	**Measure**	**Revised Estimate (95% CI)**	**P-value**	**I², %**	**Direction Change**
**BCTQ-SSS**	Suppaphol (2012)	4	MD	−0.42 (−1.44 to 0.60)	0.28	98.6	No
	Castillo (2014)	4	MD	−0.36 (−1.36 to 0.65)	0.34	98.6	No
	Tarallo (2014)	4	MD	−0.01 (−0.05 to 0.02)	0.41	0.0	No
	Zhang (2016)	4	MD	−0.46 (−1.71 to 0.80)	0.33	98.6	No
	Gupta (2025)	4	MD	−0.46 (−1.73 to 0.82)	0.34	98.0	No
**BCTQ-FSS**	Suppaphol (2012)	4	MD	−0.29 (−0.99 to 0.41)	0.28	95.5	No
	Castillo (2014)	4	MD	−0.31 (−0.95 to 0.34)	0.23	95.5	No
	Tarallo (2014)	4	MD	−0.10 (−0.27 to 0.06)	0.15	30.0	No
	Zhang (2016)	4	MD	−0.34 (−1.08 to 0.40)	0.24	94.4	No
	Gupta (2025)	4	MD	−0.29 (−1.16 to 0.59)	0.38	95.2	No
**Scar Tenderness (early)**	Jugovac (2002)	2	OR	0.21 (0.00–65.82)	0.18	0.0	No
	Bhattacharya (2004)	2	OR	0.24 (0.00–142.2)	0.21	0.0	No
	Hamed (2009)	2	OR	0.27 (0.00–89.81)	0.21	0.0	No
**Return to Work**	Heidarian (2013)	2	MD	−11.18 (−41.20 to 18.84)	0.13	75.8	No
	Zhang (2016)	2	MD	−8.69 (−16.47 to −0.92)	0.04	0.0	No
	Gupta (2025)	2	MD	−10.75 (−47.33 to 25.83)	0.17	83.5	No
**Incision Length**	Jugovac (2002)	2	MD	−1.67 (−6.68 to 3.35)	0.15	97.5	No
	Suppaphol (2012)	2	MD	−2.36 (−6.42 to 1.70)	0.09	89.7	No
	Heidarian (2013)	2	MD	−1.98 (−11.06 to 7.11)	0.22	97.9	No
**Operative Time**	Jugovac (2002)	2	MD	−2.94 (−35.97 to 30.09)	0.46	96.5	No
	Suppaphol (2012)	2	MD	−3.53 (−28.30 to 21.25)	0.32	97.8	No
	Heidarian (2013)	2	MD	−1.18 (−8.90 to 6.54)	0.30	55.7	No
**Pillar Pain (late)**	Hamed (2009)	3	OR	1.22 (0.17 to 8.99)	0.81	20.7	No
	Larsen (2013)	3	OR	0.92 (0.03 to 25.12)	0.94	70.4	No
	Zhang (2016)	3	OR	0.59 (0.03 to 11.09)	0.65	62.8	No
	Tarallo (2014)	3	OR	0.57 (0.04 to 8.95)	0.60	58.2	No
**Grip Strength**	Hamed (2009)	2	SMD	0.27 (−10.51 to 11.05)	0.75	89.6	No
	Suppaphol (2012)	2	SMD	−0.01 (−6.99 to 6.98)	1.00	79.4	No
	Castillo (2014)	2	SMD	0.78 (−2.97 to 4.53)	0.31	27.5	No
**Panel B - Outlier and Influence Diagnostics:**							
**Outcome**	**Study**	**Weight, %**	**Studentized Residual**	**Cook's Distance**	**Q Contribution**	**Outlier^a^**	**Influential^b^**
**BCTQ-SSS**	Suppaphol (2012)	20.9	0.51	0.145	0.20	No	No
	Castillo (2014)	12.7	−0.03	0.000	0.00	No	No
	Tarallo (2014)	21.8	−2.05	2.428	3.28	Yes	Yes
	Zhang (2016)	22.1	0.79	0.363	0.48	No	No
	Gupta (2025)	22.4	0.77	0.354	0.46	No	No
**BCTQ-FSS**	Suppaphol (2012)	19.9	0.30	0.104	0.07	No	No
	Castillo (2014)	11.4	0.63	0.290	0.35	No	No
	Tarallo (2014)	22.2	−2.04	5.241	3.23	Yes	Yes
	Zhang (2016)	23.0	0.89	1.020	0.61	No	Yes
	Gupta (2025)	23.5	0.37	0.176	0.10	No	No
**Scar Tenderness (early)**	Jugovac (2002)	28.0	0.48	0.105	0.16	No	No
	Bhattacharya (2004)	41.9	−0.02	0.000	0.00	No	No
	Hamed (2009)	30.1	−0.45	0.097	0.14	No	No
**Return to Work**	Heidarian (2013)	41.0	1.06	0.067	0.66	No	No
	Zhang (2016)	18.2	−1.77	0.116	2.56	No	No
	Gupta (2025)	40.8	0.33	0.007	0.07	No	No
**Incision Length**	Jugovac (2002)	31.9	−1.31	0.925	1.17	No	No
	Suppaphol (2012)	34.0	1.42	1.115	1.33	No	No
	Heidarian (2013)	34.1	−0.13	0.009	0.01	No	No
**Operative Time**	Jugovac (2002)	34.7	0.45	0.007	0.13	No	No
	Suppaphol (2012)	31.6	1.03	0.036	0.72	No	No
	Heidarian (2013)	33.7	−1.46	0.075	1.42	No	No
**Pillar Pain (late)**	Hamed (2009)	25.0	−1.42	0.669	1.41	No	No
	Larsen (2013)	25.8	−0.46	0.075	0.15	No	No
	Zhang (2016)	24.6	0.88	0.254	0.55	No	No
	Tarallo (2014)	24.5	1.01	0.334	0.72	No	No
**Grip Strength**	Hamed (2009)	34.9	0.26	0.036	0.04	No	No
	Suppaphol (2012)	32.3	1.12	0.600	0.81	No	No
	Castillo (2014)	32.8	−1.38	0.929	1.23	No	No
**Panel C - Pre-Specified Sensitivity Analyses:**							
**Sensitivity Analysis**		**Outcome**	**k**	**Measure**	**Revised Estimate (95% CI)**	**P-value**	**I², %**
Excluding Tarallo et al. 2014 (outlier)		BCTQ-SSS	4	MD	−0.01 (−0.05 to 0.02)	0.41	0.0
Excluding Tarallo et al. 2014 (outlier)		BCTQ-FSS	4	MD	−0.10 (−0.27 to 0.06)	0.15	30.0
Excluding Bhattacharya et al. 2004 (paired design)		Scar Tenderness (early)	2	OR	0.24 (0.00–142.2)	0.21	0.0
Excluding Larsen et al. 34 (endoscopic technique)		Pillar Pain (late)	3	OR	0.92 (0.03–25.12)	0.92	70.4
Excluding Heidarian et al. 2013 (Knifelight, short FU)		Return to Work	2	MD	−11.18 (−41.20 to 18.84)	0.13	75.8
Restricted to FU ≥12 months		BCTQ-SSS	2	MD	−0.25 (−3.43 to 2.93)	0.50	97.2
Restricted to FU ≥12 months		BCTQ-FSS	2	MD	−0.20 (−2.75 to 2.34)	0.49	96.6
**Panel D - Univariate Meta-Regression:**							
**Outcome**	**Moderator**	**β Coefficient**	**95% CI**	**P-value**	**R², %**	**Residual I², %**	**Q_E_**
**BCTQ-SSS**	Publication year	0.0442	0.0347 to 0.0537	0.003	37.9	97.8	136.65
	Total sample size	−0.0050	−0.0064 to −0.0035	0.006	21.1	98.3	172.86
	MI incision length (cm)	−0.3017	−0.4236 to −0.1798	0.02	10.5	98.5	195.85
	Follow-up (months)	−0.0016	−0.0064 to 0.0031	0.55	0.0	98.6	218.93
**BCTQ-FSS**	Publication year	0.0172	0.0045 to 0.0299	0.08	9.6	95.0	59.45
	Total sample size	−0.0010	−0.0027 to 0.0008	0.35	0.3	95.4	65.24
	MI incision length (cm)	0.0475	−0.1047 to 0.1998	0.58	0.0	95.5	66.08
	Follow-up (months)	0.0081	0.0025 to 0.0136	0.07	11.4	94.9	58.35

**Notes:** Panel A reports the pooled effect estimate and I² after sequentially removing each study from the meta-analysis; direction change indicates whether the point estimate reversed relative to the overall pooled result; Panel B reports influence diagnostics for each study within each meta-analysis; ^a^outlier defined as absolute studentized residual > 2.0; ^b^influential defined as Cook's distance exceeding 4/k threshold; Tarallo et al. 2014 was flagged as both an outlier and influential for BCTQ-SSS and BCTQ-FSS with its removal reducing I² from 98.2% to 0.0% and from 94.0% to 30.0% respectively; Panel C reports pre-specified sensitivity analyses testing robustness under clinically motivated exclusions; Panel D reports univariate random-effects meta-regression (method of moments) for BCTQ-SSS and BCTQ-FSS as these were the only outcomes with k ≥ 5; β represents the change in pooled MD per one-unit increase in the moderator; R² is the proportion of between-study variance (τ²) explained by the moderator; publication year was a significant moderator for SSS (P = 0.003, R² = 37.9%) suggesting that more recent studies show smaller MI advantages. **Abbreviations:** BCTQ = Boston Carpal Tunnel Questionnaire; CI = Confidence Interval; FSS = Functional Status Scale; FU = Follow-up; I² = Higgins inconsistency index; k = number of studies; MD = Mean Difference; MI = Minimally Invasive; OR = Odds Ratio; Q_E_ = residual heterogeneity statistic; R² = proportion of heterogeneity explained; SSS = Symptom Severity Scale; β = meta-regression coefficient; τ² = between-study variance.

### Publication bias

**Fig. 4:**
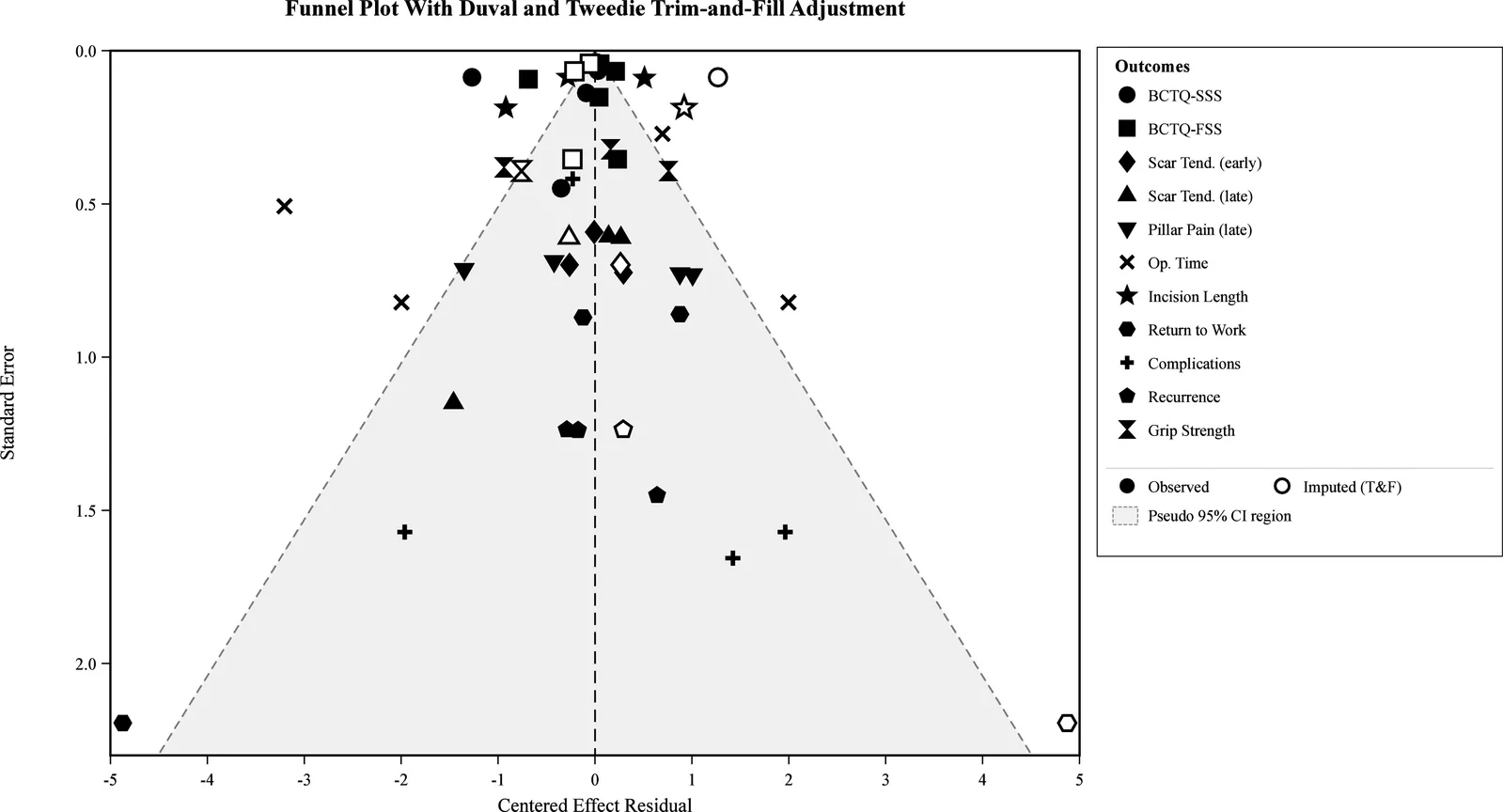
Funnel plot with trim-and-fill adjustment for publication bias assessment.

No outcome showed significant asymmetry on Egger's/Peters' tests and through visual inspection of funnel plot of asymmetry for publication bias assessment **(Supplementary Table 3;** Fig. 4). However, the Rücker limit meta-analysis detected small-study effects for BCTQ-SSS (P-value<0.001), BCTQ-FSS (P-value = 0.08), operative time (P-value = 0.07), incision length (P-value<0.001), and return to work (P-value = 0.02). The bias-adjusted BCTQ-SSS estimate shifted from −0.36 to +0.04 (95% CI: 0.01 to 0.06), crossing the null and indicating the MI advantage was attributable to small-study effects and the Tarallo outlier **(Supplementary Table 3)**.

### Trial sequential analysis

**Fig. 5:**
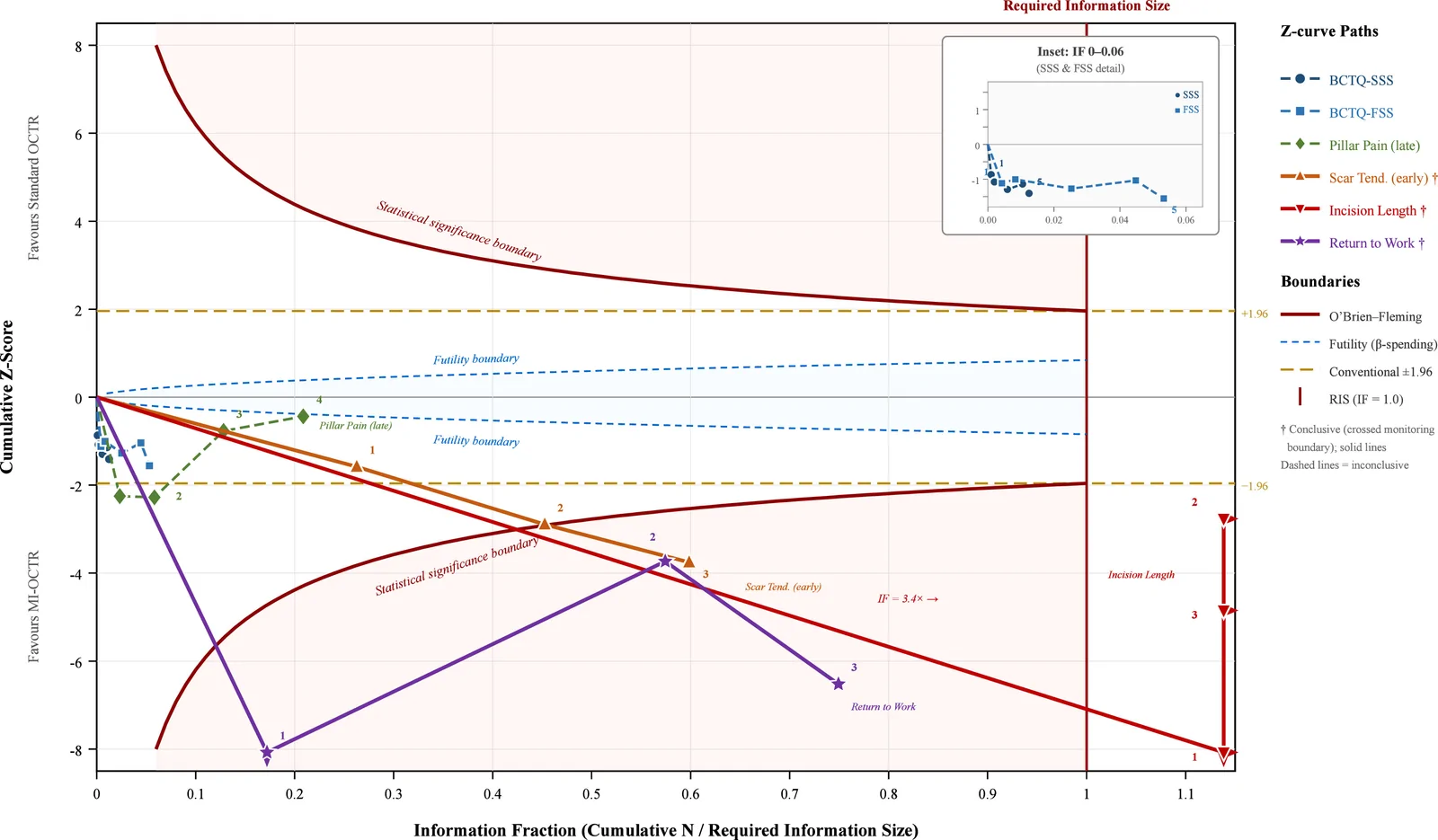
Trial sequential analysis plot for outcomes.

For BCTQ-SSS, only 1.2% of the RIS (30,276) had been accrued, and for BCTQ-FSS, only 5.3% (RIS = 7081), with both remaining inconclusive **(Supplementary Table 2, Panel B;** Fig. 5). Late pillar pain (20.9% of RIS) was also inconclusive. Three outcomes crossed the monitoring boundary for conclusive benefit: scar tenderness early (60.0% of RIS; TSA-adjusted 95% CI: 0.09 to 0.63), incision length (342.6% of RIS; TSA-adjusted 95% CI: −2.38 to −1.61), and return to work (74.9% of RIS; TSA-adjusted 95% CI: −12.28 to −7.05) (Fig. 5).

### Certainty of evidence

The certainty was very low for most outcomes, including BCTQ-SSS, BCTQ-FSS, pillar pain, operative time, incision length, grip strength, return to work, complications, nerve injury, recurrence, and scar hypertrophy (Table 5). Early and late scar tenderness were the only outcomes rated low certainty. Downgrading was driven by serious risk of bias, very serious inconsistency for primary outcomes, serious imprecision, and serious indirectness when pooling across heterogeneous MI techniques (Table 5).

**Table 5:** GRADE summary of findings.

**Outcome**	**k**	**Participants (MI / Std)**	**Effect Estimate (95% CI)**	**Risk of Bias**	**Inconsistency**	**Indirectness**	**Imprecision**	**Publication Bias**	**Certainty of Evidence**
BCTQ-SSS	5	191/186	−0.36 (−1.07 to 0.35)	Serious^a^	Very serious^b^	Not serious	Serious^c^	Undetected	⊕°°° Very Low
BCTQ-FSS	5	191/186	−0.27 (−0.75 to 0.21)	Serious^a^	Very serious^b^	Not serious	Serious^c^	Undetected	⊕°°° Very Low
Pillar Pain (early ≤6 wk)	2	45/47	0.36 (0.00–23,744.4)	Serious^a^	Serious^d^	Not serious	Very serious^e^	Undetected	⊕°°° Very Low
Pillar Pain (late ≥3 mo)	4	182/176	0.78 (0.13–4.65)	Serious^a^	Serious^d^	Serious^f^	Serious^c^	Undetected	⊕°°° Very Low
Scar Tenderness (early ≤3 mo)	3	81/83	0.24 (0.05–1.23)	Serious^a^	Not serious	Not serious	Serious^c^	Undetected	⊕⊕°° Low
Scar Tenderness (late ≥6 mo)	3	152/146	0.77 (0.14–4.31)	Serious^a^	Not serious	Not serious	Serious^c^	Undetected	⊕⊕°° Low
Operative Time	3	80/81	−2.50 (−9.20 to 4.19)	Serious^a^	Very serious^b^	Serious^g^	Serious^c^	Undetected	⊕°°° Very Low
Incision Length	3	80/81	−2.00 (−3.77 to −0.22)	Serious^a^	Very serious^b^	Serious^g^	Not serious	Undetected	⊕°°° Very Low
Grip Strength	3	47/52	0.36 (−1.76 to 2.47)	Serious^a^	Serious^d^	Not serious	Serious^c^	Undetected	⊕°°° Very Low
Return to Work	3	132/125	−9.66 (−16.04 to −3.29)	Serious^a^	Serious^d^	Serious^g^	Not serious	Undetected	⊕°°° Very Low
Total Complications	3	116/116	1.04 (0.06–17.54)	Serious^a^	Not serious	Serious^f^	Very serious^e^	Undetected	⊕°°° Very Low
Nerve Injury	2	49/51	4.37 (0.00–8777,169.0)	Serious^a^	Not serious	Serious^f^	Very serious^e^	Undetected	⊕°°° Very Low
Symptom Recurrence	3	152/146	0.58 (0.02–14.72)	Serious^a^	Not serious	Serious^f^	Serious^c^	Undetected	⊕°°° Very Low
Scar Hypertrophy	2	90/90	0.11 (0.00–96,929.2)	Serious^a^	Not serious	Not serious	Very serious^e^	Undetected	⊕°°° Very Low

**Notes:** Certainty of evidence rated using the GRADE framework starting at High for randomized controlled trials and downgraded as appropriate across five domains; ^a^most included trials had unclear allocation concealment and none achieved participant or surgeon blinding due to the nature of surgical interventions, several had unclear or absent assessor blinding; ^b^I² > 90% with prediction interval crossing null, Tarallo et al. 2014 identified as outlier and sole driver of heterogeneity via influence diagnostics (studentized residual > 2.0, Cook's distance > 4/k); ^c^95% CI crosses the null and/or does not exclude clinically meaningful effects in either direction, total sample size below optimal information size; ^d^I² 50–75% indicating moderate-to-significant heterogeneity; ^e^k = 2–3 with very wide CI and very few events, total information fraction far below optimal information size; ^f^pooled across heterogeneous MI technique types (Mini-Open, Knifelight, Endoscopic) with different mechanisms, and/or varied complication definitions across studies; ^g^pooled across heterogeneous MI technique types (Mini-Open and Knifelight) with heterogeneous measurement methods and/or includes return-to-ADL mixed with return-to-work endpoints. **Abbreviations:** CI = Confidence Interval; GRADE = Grading of Recommendations Assessment, Development, and Evaluation; I² = Higgins inconsistency index; k = number of studies; MD = Mean Difference; MI = Minimally Invasive; OR = Odds Ratio; SMD = Standardized Mean Difference; Std = Standard Open; ⊕ = met quality criterion; ° = did not meet quality criterion.

## Discussion

Our study represents the first structured meta-analysis with TSA, to our knowledge, investigating the full spectrum of MI-OCTR techniques against standard open CTR. The principal findings demonstrated that MI-OCTR and standard open CTR resulted in comparable efficacy in symptom relief, as measured by the BCTQ-SSS (MD = −0.36, P-value = 0.23) and BCTQ-FSS (MD = −0.27, P-value = 0.19), with neither outcome reaching statistical significance. MI-OCTR was associated with significantly shorter incision length (MD = −2.00 cm, P-value = 0.04) and earlier return to work (MD = −9.66 days, P-value = 0.02), while safety outcomes including complications, nerve injury, recurrence, and scar hypertrophy were equivalent between techniques. However, very high heterogeneity (I² = 98.2% for BCTQ-SSS), very low GRADE certainty for most outcomes, and TSA indicating that only 1.2% of the required information size had been accrued collectively suggest that the current evidence base remains insufficient for definitive conclusions.

The observed equivalence in patient-reported outcomes between MI-OCTR and standard open CTR aligns with the broader body of literature comparing minimally invasive and conventional approaches. Sayegh and Strauch,^24^ in a meta-analysis of 21 RCTs including 1859 hands, reported similar BCTQ scores between endoscopic and open release, with outcome differences dissipating beyond six months of follow-up. Li et al.,^11^ in an expanded analysis of 28 RCTs, similarly found no significant differences in BCTQ-SSS or BCTQ-FSS between endoscopic and open CTR. Our findings extend this pattern to the specific comparison of MI-OCTR techniques, which include mini-open, Knifelight, and limited-incision approaches that have been less only investigated as a collective category. The return-to-work advantage observed in our analysis (MD = −9.66 days) was of greater magnitude than that reported by Li et al. for endoscopic versus open CTR (MD = −7.25 days, P-value = 0.04), which may reflect the practical benefits of shorter palmar incisions that spare the overlying soft tissue structures more effectively than the standard longitudinal approach.^11^

An important distinction between our findings and the endoscopic CTR literature concerns safety. Sayegh and Strauch^24^ demonstrated that endoscopic release was associated with a significantly elevated risk of nerve injury (RR = 2.84, 95% CI: 1.08 to 7.46, P-value = 0.03), a finding corroborated by Li et al.^11^ who reported significantly higher transient nerve injury rates with endoscopic CTR (OR = 4.87, 95% CI: 1.37 to 17.25, P-value = 0.01). The Cochrane-affiliated analysis by Vasiliadis et al.^12^ also characterized the elevated nerve injury risk as an inherent concern of the endoscopic approach. In contrast, our analysis demonstrated no significant differences in nerve injury (OR = 4.37, P-value = 0.42) or any other safety endpoint between MI-OCTR and standard open CTR, with pooled 95% CIs including the null for all safety outcomes. This suggests that MI-OCTR techniques may offer the perioperative advantages of minimally invasive surgery, particularly regarding scar morbidity and return to work, without introducing the elevated nerve injury risk that has been consistently reported with endoscopic approaches. However, this interpretation warrants caution given the limited number of studies reporting safety data (k = 2–3 per outcome), very wide HKSJ-adjusted 95% CIs, and very low GRADE certainty.

The identification of Tarallo et al.^25^ as a sole outlier driving virtually all heterogeneity in primary outcomes represents a significant finding that warrants careful discussion. Removing this single study reduced I² from 98.2% to 0.0% for BCTQ-SSS and from 94.0% to 30.0% for BCTQ-FSS, with influence diagnostics confirming its outsized impact (Cook's distances: 2.428 and 5.241). Meta-regression further demonstrated that publication year was a significant moderator (β = 0.0442, P-value = 0.003, R² = 37.9%), indicating that newer studies reported progressively smaller differences between MI-OCTR and standard open CTR. This temporal attenuation pattern may reflect improvements in standard open technique over time, regression to the mean, or methodological refinements in outcome measurement. Regardless of the underlying mechanism, the convergence of effect estimates toward the null in more recent trials raises important concerns about whether early studies may have overestimated the MI-OCTR advantage.

From a clinical significance perspective, the pooled BCTQ-SSS difference of −0.36 points falls well below the established MCID for the symptom severity scale, which has been reported as 1.04 points by Ozyurekoglu et al.^26^ and 1.14 points by Kim and Jeon.^27^ Even under the most liberal MCID threshold of 0.64 points proposed by Fernández-de-las-Peñas et al.,^28^ the observed pooled difference does not reach clinical relevance. In addition to that, the 95% prediction interval (−1.93 to 1.22) crossing the null indicates that a future study could plausibly demonstrate effects in either direction, further underscoring the uncertainty surrounding the true between-technique difference. These findings suggest that, even if sufficient statistical power were eventually achieved, the absolute magnitude of any MI-OCTR advantage in symptom relief is unlikely to be clinically meaningful.

The TSA findings represent one of the most concerning aspects of the current evidence base. With only 1.2% of the required information size accrued for BCTQ-SSS (377 of 30,276 needed participants) and 5.3% for BCTQ-FSS, the evidence for primary outcomes is significantly underpowered. Of the six outcomes evaluated by TSA, only three crossed the monitoring boundary: early scar tenderness, incision length, and return to work. The remaining outcomes, including both primary BCTQ scales and late pillar pain, remained inconclusive, which means that the current non-significant pooled estimates cannot be interpreted as evidence of equivalence. These results parallel the Rücker limit meta-analysis findings, which detected significant small-study effects for BCTQ-SSS (P-value<0.001) and demonstrated that the bias-adjusted estimate shifted from −0.36 to +0.04 (95% CI: 0.01 to 0.06), crossing the null and suggesting that the apparent MI-OCTR advantage was at least partially attributable to publication bias.

A recent best-evidence systematic review and meta-analysis by Hammert et al.,^29^ investigating 23 prospective studies with 2303 mini-open CTR patients, reported statistically significant and clinically important improvements in BCTQ-SSS (MD = −2.2, P-value<0.001) and BCTQ-FSS (MD = −2.1, P-value<0.001) following surgery, with meta-regression demonstrating that complication risk increased with longer incision lengths (P-value = 0.008). This dose-response relationship between incision length and morbidity provides biological plausibility for our finding that MI-OCTR achieved significantly shorter incisions (MD = −2.00 cm), which may explain part of the borderline scar tenderness reduction observed in the early postoperative period. However, it should be noted that the Hammert et al.^29^ analysis was designed to evaluate pre-to-post surgical improvement, whereas our study investigated the between-group comparative effect, which represents a different question.

Several limitations of our study should be acknowledged for further consideration in future studies. First, the small number of included RCTs (k = 10) and limited total sample size (n = 660) limited statistical power, especially for subgroup analyses and meta-regression. Second, inherent limitations in blinding for surgical trials comparing visually different incision approaches contributed to the high risk of bias observed in six of ten studies. Third, the heterogeneity of MI-OCTR techniques pooled together, including mini-open, Knifelight, and endoscopic approaches, may have introduced clinical heterogeneity, although subgroup analyses demonstrated no significant interactions between technique types. Fourth, the follow-up period varied across studies (three weeks to 36 months), and the limited number of studies reporting each outcome restricted the applicability of certain analyses. In addition to these, only five of ten studies reported BCTQ outcomes, further constraining the power of primary efficacy analyses.

The current evidence suggests that MI-OCTR and standard open CTR provide similar symptom relief, with MI-OCTR offering possible perioperative advantages in incision length and return to work without additional safety concerns. However, the evidence base is critically underpowered, with fewer than 2% of required participants enrolled for the primary outcome. The very low GRADE certainty for most outcomes, significant influence of a single outlier study, and detectable publication bias collectively indicate that these findings should be interpreted with caution. Adequately powered, multicenter RCTs with standardized MI-OCTR definitions and BCTQ as the primary endpoint are needed to determine whether MI-OCTR techniques offer meaningful clinical advantages over standard open CTR for patients with CTS.

## Conclusions

This meta-analysis of ten RCTs including 660 participants demonstrated that MI-CTR had comparable long-term functional and symptomatic outcomes to standard open release while offering distinct short-term advantages. Based on our analyses, we found that MI-CTR was associated with significantly reduced scar tenderness, decreased pillar pain at three to six months, and earlier return to work and daily activities. Complication rates were similar between approaches, supporting the safety profile of minimally invasive techniques. These findings suggest that MI-CTR represents a viable alternative to conventional OCTR, especially for patients in whom expedited functional recovery is a clinical priority. However, heterogeneity in surgical techniques and outcome measures across the included RCTs limits definitive conclusions. Future large-scale, standardised RCTs with uniform definitions of minimally invasive approaches and long-term follow-up beyond 12 months are warranted to guide evidence-based surgical decision-making.

## Funding

N/A

## Ethical Approval

N/A

## Conflict of Interest

N/A.

## CRediT authorship contribution statement

**Ahmed Khaled Almarri:** Conceptualization, Methodology, Investigation, Formal analysis, Writing – original draft, Project administration. **Yazan J Alalwani:** Conceptualization, Methodology, Formal analysis, Writing – original draft. **Ammar Alsayegh:** Validation, Formal analysis, Writing – review & editing. **Mohammed Khder M. Alamri:** Validation, Formal analysis, Writing – review & editing. **Faisal Falah Alanazi:** Data curation, Investigation, Writing – review & editing. **Abdulmalik Hadi AlEnazy:** Methodology, Software, Visualization. **Daniyah Adil A. Alharbi:** Methodology, Software, Visualization. **Nawaf Salman Almutairi:** Resources, Data curation, Writing – review & editing. **Ghayed Ahmad AlWassiah:** Formal analysis, Writing – review & editing. **Anna Abdulmohsen Almutlaq:** Formal analysis, Writing – review & editing. **Abdulmajeed Abdulaziz Alfouzan:** Methodology, Investigation, Writing – review & editing. **Fadi Ibrahim Alhobayyeb:** Resources, Validation, Writing – review & editing. **Raneem M. Turkistani:** Data curation, Investigation, Writing – review & editing. **Abdulaziz Ibrahim Alzahrani:** Data curation, Investigation, Writing – review & editing. **Ahmed Y Azzam:** Conceptualization, Supervision, Formal analysis, Writing – review & editing, Project administration.
