# Supplementary material for: Minimally invasive vs standard open carpal tunnel release: A systematic review, meta-analysis, and trial sequential analysis of randomized controlled trials
Source: JPRAS Open. 2026 Sep 11;52:375–89. doi: 10.1016/j.jpra.2026.08.018 (PMC13584054; doi:10.1016/j.jpra.2026.08.018)
Supplement: Supplementary file 1 [file mmc1.docx]

**Supplementary Table 1:** Risk of Bias Assessment Using the Cochrane Risk of Bias 2 (RoB 2) Tool.

| **Study** | **D1: Randomization Process** | **D2: Deviations from Intended Interventions** | **D3: Missing Outcome Data** | **D4: Measurement of the Outcome** | **D5: Selection of the Reported Result** | **Overall** | **Overall Rationale** |
| --- | --- | --- | --- | --- | --- | --- | --- |
| Gupta et al. 2025 | + Low | − High | + Low | ? Some concerns | + Low | **− High** | High risk in D2 (unblinded surgical trial) |
| Zhang et al. 2016 | ? Some concerns | − High | + Low | ? Some concerns | ? Some concerns | **− High** | High risk in D2; some concerns in D1, D4, D5 |
| Tarallo et al. 2014 | ? Some concerns | − High | + Low | ? Some concerns | ? Some concerns | **− High** | High risk in D2; some concerns in D1, D4, D5 |
| Castillo and Yao 2014 | + Low | ? Some concerns | ? Some concerns | + Low | + Low | **? Some concerns** | Some concerns in D2 (surgeon unblinded) and D3 (moderate attrition) |
| Heidarian et al. 2013 | ? Some concerns | − High | + Low | − High | ? Some concerns | **− High** | High risk in D2 and D4 |
| Larsen et al. 2013 | + Low | ? Some concerns | ? Some concerns | ? Some concerns | ? Some concerns | **? Some concerns** | Some concerns across D2, D3, D4, D5; no single high-risk domain |
| Suppaphol et al. 2012 | ? Some concerns | − High | + Low | ? Some concerns | ? Some concerns | **− High** | High risk in D2; some concerns in D1, D4, D5 |
| Hamed et al. 2009 | + Low | ? Some concerns | + Low | + Low | + Low | **? Some concerns** | Some concerns in D2 only (surgeon unblinded; inherent to surgical trials) |
| Bhattacharya et al. 2004 | ? Some concerns | ? Some concerns | + Low | ? Some concerns | ? Some concerns | **? Some concerns** | Some concerns in D1, D2, D4, D5; split-body design mitigates some bias |
| Jugovac et al. 2002 | ? Some concerns | − High | + Low | ? Some concerns | ? Some concerns | **− High** | High risk in D2; some concerns in D1, D4, D5 |

***Notes:*** *Risk of bias was assessed using the Cochrane Risk of Bias 2 (RoB 2) tool for individually randomized parallel-group trials; each domain was judged as low risk of bias (+), some concerns (?), or high risk of bias (−); overall judgment followed the RoB 2 algorithm where high risk in any single domain results in an overall high-risk rating and some concerns in multiple domains without any high-risk domain results in an overall some-concerns rating; D2 was rated high risk in six of ten studies because blinding of participants and surgeons is inherently impossible in open surgical trials comparing visibly different incision approaches; the three studies rated as some concerns overall (Castillo and Yao 2014, Larsen et al. 2013, Hamed et al. 2009) achieved patient blinding through standardized dressings and utilized independent blinded assessors; Bhattacharya et al. 2004 used a within-patient split-body design which inherently reduces performance bias and was therefore rated some concerns rather than high for D2; D3 was rated low risk in eight of ten studies; Castillo and Yao 2014 and Larsen et al. 2013 were rated some concerns due to moderate attrition; the summary row presents the proportion of studies at each risk level per domain.* ***Abbreviations:*** *D1 = Domain 1; D2 = Domain 2; D3 = Domain 3; D4 = Domain 4; D5 = Domain 5; RoB 2 = Cochrane Risk of Bias 2 tool; + = Low risk of bias; ? = Some concerns; − = High risk of bias.*
