# Supplementary material for: Minimally invasive vs standard open carpal tunnel release: A systematic review, meta-analysis, and trial sequential analysis of randomized controlled trials
Source: JPRAS Open. 2026 Sep 11;52:375–89. doi: 10.1016/j.jpra.2026.08.018 (PMC13584054; doi:10.1016/j.jpra.2026.08.018)
Supplement: Supplementary file 2 [file mmc2.docx]

**Supplementary Table 2:** Cumulative Meta-Analysis and Trial Sequential Analysis.

| **Panel A - Cumulative Meta-Analysis (Studies Added Chronologically):** | | | | | | | | |
| --- | --- | --- | --- | --- | --- | --- | --- | --- |
| **Outcome** | **Step** | **Study Added** | **k** | **Cumulative N** | **Cumulative Estimate (95% CI)** | **P-value** | **I², %** | **Statistically Significant** |
| **BCTQ-SSS** | 1 | Suppaphol et al. 2012 | 1 | 30 | -0.12 (-0.39 to 0.15) | 0.38 | — | No |
|  | 2 | Castillo and Yao 2014 | 2 | 59 | -0.14 (-1.82 to 1.53) | 0.48 | 0 | No |
|  | 3 | Tarallo et al. 2014 | 3 | 179 | -0.62 (-2.69 to 1.45) | 0.33 | 96.3 | No |
|  | 4 | Zhang et al. 2016 | 4 | 317 | -0.46 (-1.73 to 0.82) | 0.34 | 98.0 | No |
|  | 5 | Gupta et al. 2025 | 5 | 377 | -0.36 (-1.07 to 0.35) | 0.23 | 98.2 | No |
| **BCTQ-FSS** | 1 | Suppaphol et al. 2012 | 1 | 30 | -0.17 (-0.47 to 0.13) | 0.26 | — | No |
|  | 2 | Castillo and Yao 2014 | 2 | 59 | -0.14 (-1.91 to 1.63) | 0.50 | 0 | No |
|  | 3 | Tarallo et al. 2014 | 3 | 179 | -0.40 (-1.74 to 0.95) | 0.33 | 90.4 | No |
|  | 4 | Zhang et al. 2016 | 4 | 317 | -0.28 (-1.16 to 0.59) | 0.38 | 95.2 | No |
|  | 5 | Gupta et al. 2025 | 5 | 377 | -0.27 (-0.75 to 0.21) | 0.19 | 94.0 | No |
| **Pillar Pain (late)** | 1 | Hamed et al. 2009 | 1 | 40 | 0.20 (0.05–0.81) | 0.02 | — | Yes |
|  | 2 | Larsen et al. 2013 | 2 | 100 | 0.32 (0.00–176.8) | 0.26 | 0 | No |
|  | 3 | Tarallo et al. 2014 | 3 | 220 | 0.59 (0.03–11.09) | 0.52 | 62.8 | No |
|  | 4 | Zhang et al. 2016 | 4 | 358 | 0.78 (0.13–4.65) | 0.69 | 58.8 | No |
| **Scar Tenderness (early)** | 1 | Jugovac et al. 2002 | 1 | 72 | 0.32 (0.08–1.31) | 0.11 | — | No |
|  | 2 | Bhattacharya et al. 2004 | 2 | 124 | 0.27 (0.00–89.81) | 0.21 | 0 | No |
|  | 3 | Hamed et al. 2009 | 3 | 164 | 0.24 (0.05–1.23) | 0.06 | 0 | No |
| **Incision Length** | 1 | Jugovac et al. 2002 | 1 | 72 | −2.70 (−3.07 to −2.33) | <0.001 | — | Yes |
|  | 2 | Suppaphol et al. 2012 | 2 | 102 | −1.98 (−11.06 to 7.11) | 0.22 | 97.9 | No |
|  | 3 | Heidarian et al. 2013 | 3 | 161 | −2.00 (−3.77 to −0.22) | 0.04 | 97.0 | Yes |
| **Return to Work** | 1 | Heidarian et al. 2013 | 1 | 59 | −8.20 (−9.89 to −6.51) | <0.001 | — | Yes |
|  | 2 | Zhang et al. 2016 | 2 | 197 | −10.75 (−47.33 to 25.83) | 0.17 | 83.5 | No |
|  | 3 | Gupta et al. 2025 | 3 | 257 | −9.66 (−16.04 to −3.29) | 0.02 | 67.2 | Yes |
| **Panel B - Trial Sequential Analysis:** | | | | | | | | |
| **Outcome** | **MCID** | **D², %** | **RIS** | **Accrued N** | **AIF, %** | **Conventional 95% CI** | **TSA-Adjusted 95% CI** | **Interpretation** |
| **BCTQ-SSS** | MD = 0.3 | 98.2 | 30276 | 377 | 1.2 | -0.36 (-1.07 to 0.35) | -0.36 (-4.62 to 3.90) | Inconclusive |
| **BCTQ-FSS** | MD = 0.3 | 94.0 | 7081 | 377 | 5.3 | -0.27 (-0.75 to 0.21) | -0.27 (-1.68 to 1.14) | Inconclusive |
| **Pillar Pain (late)** | OR = 0.5 | 58.8 | 1716 | 358 | 20.9 | 0.78 (0.13–4.65) | 0.78 (0.07–8.57) | Inconclusive |
| **Scar Tenderness (early)** | OR = 0.5 | 0.0 | 274 | 164 | 60.0 | 0.24 (0.05–1.23) | 0.24 (0.09–0.63) | Conclusive benefit |
| **Incision Length** | MD = 1.0 cm | 97.0 | 47 | 161 | 342.6 | −2.00 (−3.77 to −0.22) | −2.00 (−2.38 to −1.61) | Conclusive benefit |
| **Return to Work** | MD = 7.0 days | 67.2 | 343 | 257 | 74.9 | −9.66 (−16.04 to −3.29) | −9.66 (−12.28 to −7.05) | Conclusive benefit |

***Notes:*** *Panel A presents cumulative meta-analysis with studies added in chronological order of publication; at each step the pooled estimate was recalculated incorporating all studies up to that point; for BCTQ-SSS and BCTQ-FSS the cumulative estimate never reached statistical significance at any step, and I² rose sharply upon addition of Tarallo et al. 2014 (from 0% to 96.3% for SSS and from 0% to 90.4% for FSS) confirming this study as the inflection point for heterogeneity; for Pillar Pain the initially significant estimate from Hamed et al. 2009 alone (OR 0.20, P = 0.03) lost significance upon pooling with subsequent studies; Panel B reports trial sequential analysis with two-sided α = 0.05, power 80%, and clinically meaningful differences set at MD = 0.30 points on the Levine BCTQ scale for SSS and FSS and OR = 0.50 for binary outcomes; D² represents the proportion of the required information size attributable to between-study heterogeneity; RIS is the meta-analytic equivalent of a sample size calculation adjusted for heterogeneity; AIF is the proportion of RIS accrued; TSA-adjusted CIs apply O'Brien–Fleming α-spending monitoring boundaries; for BCTQ-SSS only 1.2% of the RIS has been accrued indicating that the current evidence is far from conclusive; scar tenderness (early) reached 60.0% of RIS and crossed the TSA monitoring boundary for benefit (TSA-adjusted upper CI 0.63 < 1.00).* ***Abbreviations:*** *AIF = Accrued Information Fraction; BCTQ = Boston Carpal Tunnel Questionnaire; CI = Confidence Interval; D² = diversity; FSS = Functional Status Scale; I² = Higgins inconsistency index; k = number of studies; MCID = Minimal Clinically Important Difference; MD = Mean Difference; N = total participants; OR = Odds Ratio; RIS = Required Information Size; SSS = Symptom Severity Scale; TSA = Trial Sequential Analysis; α = type I error rate.*
