# Supplementary material for: Minimally invasive vs standard open carpal tunnel release: A systematic review, meta-analysis, and trial sequential analysis of randomized controlled trials
Source: JPRAS Open. 2026 Sep 11;52:375–89. doi: 10.1016/j.jpra.2026.08.018 (PMC13584054; doi:10.1016/j.jpra.2026.08.018)
Supplement: Supplementary file 3 [file mmc3.docx]

**Supplementary Table 3:** Publication Bias Assessment and Small-Study Effects.

| **Outcome** | **k** | **Egger / Peters^†^ Test** | **Begg Rank Correlation** | **Trim-and-Fill** | **Limit Meta-Analysis (Rücker Model)** | | |
| --- | --- | --- | --- | --- | --- | --- | --- |
|  |  | **Intercept; P-value** | **τ; P-value** | **Imputed (Side); Adjusted Estimate (95% CI)** | **Bias-Adjusted Estimate (95% CI)** | **β_1_ Slope; P-value** | **Small-Study Effect** |
| BCTQ-SSS | 5 | -4.398; P = 0.37 | -0.400; P = 0.48 | 1 (right); -0.01 (-0.03 to 0.01) | 0.04 (0.01 to 0.06) | -4.398; P = <0.001 | Yes |
| BCTQ-FSS | 5 | -1.520; P = 0.73 | 0.200; P = 0.82 | 3 (left); -0.26 (-0.30 to -0.21) | -0.12 (-0.24 to 0.01) | -1.520; P = 0.08 | Yes |
| Pillar Pain (late)^†^ | 4 | -134.464; P = 0.12 | 0.667; P = 0.33 | 0; no adjustment | 0.00 (0.00–116.5) | 33.678; P = 0.10 | No |
| Scar Tenderness (early)^†^ | 3 | -49.943; P = 0.68 | 0.333; P = 1.00 | 1 (right); 0.25 (0.13–0.49) | 0.15 (0.00–655.9) | 0.681; P = 0.92 | No |
| Scar Tenderness (late)^†^ | 3 | -94.894; P = 0.41 | -0.333; P = 1.00 | 1 (right); 0.90 (0.43–1.89) | 6.02 (0.28–131.8) | -3.067; P = 0.18 | No |
| Operative Time | 3 | -2.584; P = 0.84 | 0.333; P = 1.00 | 1 (right); -1.75 (-2.16 to -1.34) | -1.36 (-2.45 to -0.26) | -2.584; P = 0.07 | Yes |
| Incision Length | 3 | -9.714; P = 0.59 | -0.333; P = 1.00 | 1 (right); -1.70 (-1.81 to -1.59) | -0.83 (-1.23 to -0.44) | -9.714; P = <0.001 | Yes |
| Grip Strength | 3 | −1.423; P = 0.96 | 0.333; P = 1.00 | 1 (left); 0.20 (−0.17 to 0.56) | 0.87 (−3.70 to 5.45) | −1.423; P = 0.83 | No |
| Return to Work | 3 | -4.011; P = 0.21 | -1.000; P = 0.33 | 1 (right); -8.75 (-9.86 to -7.63) | -5.22 (-8.65 to -1.79) | -4.011; P = 0.02 | Yes |
| Total Complications^†^ | 3 | 194.663; P = 0.37 | 0.333; P = 1.00 | 1 (left); 0.67 (0.32–1.41) | 0.30 (0.08–1.18) | 1.613; P = 0.11 | No |
| Symptom Recurrence^†^ | 3 | 50.986; P = 0.70 | 1.000; P = 0.33 | 1 (right); 0.63 (0.18–2.22) | 0.00 (0.00–1732831.0) | 4.101; P = 0.61 | No |

***Notes:*** *Egger's weighted linear regression was used for continuous outcomes (MD) and Peters' regression (marked with ^†^) for binary outcomes (OR) to avoid mathematical artifacts; no outcome reached significance for funnel plot asymmetry on Egger's/Peters' or Begg's tests at P < 0.10, though statistical power is limited with k = 3–5; Trim-and-Fill reports the number of imputed hypothetical "missing" studies, their side, and the adjusted pooled estimate; the Rücker limit meta-analysis estimates the treatment effect extrapolated to infinite sample size (SE = 0) with β_1_ quantifying the magnitude of small-study effects (significant at P < 0.10); small-study effects were detected for BCTQ-SSS (P < 0.001), BCTQ-FSS (P = 0.08), Operative Time (P = 0.07), Incision Length (P < 0.001), and Return to Work (P = 0.02); notably the bias-adjusted SSS estimate shifted from MD −0.36 to +0.04 crossing the null, indicating the MI advantage is attributable to small-study effects and the Tarallo outlier; P-curve analysis (applicable only to outcomes with k ≥ 5): BCTQ-SSS: 1/5 significant (insufficient for analysis); BCTQ-FSS: 2/5 significant, 2 with P < 0.025, binomial P = 0.50, Inconclusive.* ***Abbreviations:*** *CI = Confidence Interval; k = number of studies; MD = Mean Difference; OR = Odds Ratio; P = P-value; SE = Standard Error; β_1_ = regression slope coefficient; τ = Kendall's rank correlation coefficient.*
